# Gray’s Anatomy

**Published:** 1887-11

**Authors:** 


GRAY'S ANATOMY. Messrs. Lea Brothers & Co., Philadelphia, Pa., announce ready for publication a new American edition from the eleventh English edition of Gray's Descriptive and Surgical Anatomy.

This book has been so long in general use throughout the world and so universally approved that we read with surprise the announcement that this edition has been "thoroughly revised and re-edited with additions."

The work has hitherto been considered so perfect as to leave little room for improvement.

